# Relationships between fasting glucose levels, lifestyle factors, and metabolic parameters in Korean adults without diagnosis of diabetes mellitus

**DOI:** 10.1111/1753-0407.13238

**Published:** 2021-12-03

**Authors:** Seo Young Kang, Young Sik Kim

**Affiliations:** ^1^ International Healthcare Center Asan Medical Center Seoul Republic of Korea; ^2^ Department of Family Medicine, Asan Medical Center University of Ulsan College of Medicine Seoul Republic of Korea

**Keywords:** alcohol, blood pressure, fasting glucose level, lipid profiles, obesity, 空腹血糖水平, 血压, 血脂, 肥胖, 酒精

## Abstract

**Background:**

We investigated the associations between fasting glucose level ranges with lifestyle factors and metabolic profiles among adults without previous diagnosis of diabetes.

**Methods:**

We analyzed 13 625 adults without previous diagnosis of diabetes from the Korea National Health and Nutrition Examination Survey during 2016 to 2018. We categorized fasting glucose levels (mg/dl) as follows: <90, 90 to 99, 100 to 109, 110 to 124, and ≥125. We evaluated trends in the proportions of individuals with obesity, abdominal, obesity, current smoking, heavy drinking, and low physical activity according to these categories, and the odds for uncontrolled blood pressure (BP), low‐density lipoprotein cholesterol (LDL‐C), triglycerides (TG), and high‐density lipoprotein cholesterol (HDL‐C) for each fasting glucose level compared to a fasting glucose level of <90 mg/dl.

**Results:**

The proportions of individuals with obesity, abdominal obesity, and heavy drinking increased according to fasting glucose level (*P* for trend <.05). The odds for BP ≥140/90 mm Hg, TG ≥150 mg/dl, HDL‐C < 40 mg/dl in men, and HDL‐C < 50 mg/dl in women increased with increasing fasting glucose levels; however, the odds for LDL‐C ≥ 130 mg/dl increased with increasing fasting glucose levels only in women. The increases in odds for uncontrolled BP and lipid profiles were mostly observed for fasting glucose levels ≥90 mg/dl.

**Conclusions:**

Efforts are needed to prevent increased fasting glucose levels, as higher levels, even within normal range, were associated with poor metabolic profiles.

## Graphical abstract

**Highlights**

- The proportions of individuals with obesity, abdominal obesity, and heavy drinking increased according to fasting glucose level.
- The risk for uncontrolled blood pressure and lipid profiles increased with increasing fasting glucose levels.
- The increases in risks for uncontrolled blood pressure and lipid profiles were mostly observed for fasting glucose levels ≥90 mg/dl.
- Efforts are needed to prevent increased fasting glucose levels, as higher levels, even within normal range, were associated with poor metabolic profiles.

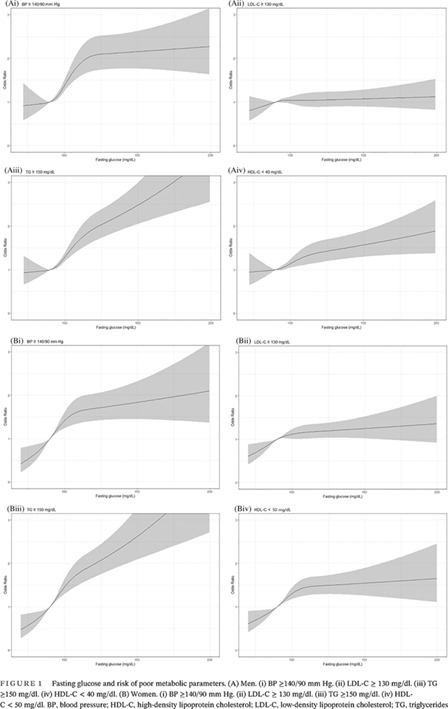

## 摘要

**背景:**

在未诊断为糖尿病的成年人中, 我们调查了空腹血糖水平范围与生活方式因素和代谢特征之间的关系

**方法:**

我们分析了2016‐2018年韩国国家健康和营养检查调查(Korea National Health And Nutrition Examination Survey)中13,625名之前未诊断糖尿病的成年人。我们将空腹血糖水平(mg/dL)分类如下:<90、90‐99、100‐109、110‐124和≥125。我们根据这些类别评估了肥胖、腹部肥胖、当前吸烟、酗酒和低体力活动的个体比例的趋势, 以及与空腹血糖水平<90 mg/dL相比, 每种空腹血糖水平不受控制的血压(BP)、低密度脂蛋白胆固醇(LDL‐C)、甘油三酯(TG)和高密度脂蛋白胆固醇(LDL‐C)的几率。

**结果:**

肥胖、腹型肥胖和大量饮酒的比例随着空腹血糖水平的升高而增加(p<0.05)。男性BP140/90 mmHg、TG≥150 mg/dL、HDL‐C<40 mg/dL和女性HDL‐C<50 mg/dL的发生几率随空腹血糖水平的升高而增加, 而LDL‐C≥130 mg/dL的发生几率仅在女性中随空腹血糖水平的升高而增加。不受控制的血压和血脂的发生几率增加主要在空腹血糖水平≥90 mg/dL时观察到。

**结论:**

需要尽可能防止空腹血糖水平升高, 因为更高的水平, 即使在正常范围内, 也与较差的代谢谱相关。

## 1 INTRODUCTION

Diabetes is increasing worldwide, with a prevalence of approximately 13.8% among adults 30 years or older in Korea.^1^, ^2^ Although diabetes is a common chronic disease, it may lead to coronary heart disease, stroke, chronic kidney disease, and blindness.^1^ Furthermore, it is a leading cause of death globally.^1^ Therefore, diabetes prevention is important to reduce the risk of further morbidities in individuals and decrease the burden on society.

The risks for diabetes, cardiovascular disease, and mortality increase in the prediabetic condition, which includes isolated impaired glucose tolerance (IGT), isolated impaired fasting glucose (IFG), the combination of IGT and IFG, and high‐risk hemoglobin A1c (HbA1c) concentrations.^3^, ^4^, ^5^ However, the progression from prediabetes to diabetes or cardiovascular disease is influenced by factors such as age, sex, ethnicity, and lifestyle.^3^, ^6^ Recently, several studies have shown prediction models for diabetes, and these models include multiple lifestyle factors and metabolic parameters as key variables.^7^, ^8^ Therefore, clinicians should understand the relationship between multiple risk factors and the prediabetic condition to develop appropriate prevention strategies.

The measurement of fasting glucose level is an easy method to screen for both prediabetes and diabetes; therefore, this measure is often evaluated in general health screening. Previous studies have shown that poor lifestyle behaviors such as heavy drinking, cigarette smoking, and low physical activity increase the risk for IFG, which, in turn, increases the risks for metabolic risk factors such as high blood pressure and dyslipidemia.^9^, ^10^, ^11^, ^12^, ^13^ However, few studies have evaluated these series of relationships in the same population. Furthermore, the association between fasting glucose levels with lifestyle risk factors and individual metabolic parameters among individuals without diabetes has received less attention, whereas these relationships in diabetes patients have been vigorously investigated.

Therefore, this study investigated the association between multiple lifestyle risk factors, fasting glucose levels, and metabolic parameters in a nationwide representative sample of adults without previous diagnosis of diabetes. We evaluated the trends of lifestyle factors and metabolic parameters according to fasting glucose levels to determine whether higher fasting glucose levels were associated with poor lifestyle behaviors and result in worse metabolic profiles in individuals who are not treated for diabetes.

## 2 METHODS

### 2.1 Participants

We used data from the seventh Korea National Health and Nutrition Examination Survey (KNHANES) (2016‐2018). The KNHANES is an annual nationwide representative cross‐sectional survey designed by the Korea Centers for Disease Control and Prevention (KCDC). The survey applies complex, stratified, clustered, and multistage probability sampling based on age, sex, and geographic areas. Further information about the study design and methodology has been reported previously.^14^ The institutional review board of the KCDC approved the study protocol, and all participants provided written informed consent before participating in the survey. Among the 24 269 participants in the seventh KNHANES, we initially included 17 036 adults aged ≥30 years. Among them, we excluded those who had been diagnosed with diabetes by a physician (*n* = 1805), taking oral hypoglycemic agents (*n* = 1666), taking insulin (*n* = 135), and with missing values for the fasting glucose levels (*n* = 1304). We additionally excluded pregnant women (*n* = 66) and those with chronic debilitating diseases such as cancer (*n* = 394), liver cirrhosis (*n* = 49), and chronic kidney disease (*n* = 51). Finally, the analysis in this study included 13 625 participants.

### 2.2 Measurements and definitions

Demographic characteristics including age, sex, household income, and educational level were collected. Household income was divided into quartiles, with the lowest quartile defined as low income. Educational level was categorized as <12 or ≥12 years. Lifestyle factors including smoking status, alcohol consumption, and physical activity were also collected. Smoking status was categorized into either current smoking or nonsmoking. Alcohol consumption was categorized according to the definitions of the National Institute on Alcohol Abuse and Alcoholism,^15^ in which heavy drinking was defined as >14, >7, and >3 standard glasses per week for men aged <65 years, men aged ≥65 years or women aged <65 years, and women aged ≥65 years, respectively. Physical activity was evaluated using the Korean version of the Global Physical Activity Questionnaire.^16^

Participant height and weight were measured using standardized techniques and equipment. Height was measured to the nearest 0.1 cm in the erect position using a portable anthropometry device. Weight was measured to the nearest 0.1 kg on a balanced scale with the participants wearing light clothing. Body mass index (BMI) was calculated by dividing the body weight by the square of height (kg/m^2^). Waist circumference was measured to the nearest 0.1 cm at the midpoint between the lowest rib and the top of the iliac crest. Systolic blood pressure (SBP) and diastolic blood pressure (DBP) were measured according to standardized methods using a sphygmomanometer while the participants were sitting. Blood pressure was measured three times at 5‐minutes intervals, with the average of the second and third measurements used in the analysis. Blood samples were collected after fasting for at least 8 hours, and biochemical values, including levels of fasting glucose, HbA1c, total cholesterol (TC), low‐density lipoprotein cholesterol (LDL‐C), triglycerides (TG), and high‐density lipoprotein cholesterol (HDL‐C), were evaluated in a certified laboratory (Hitachi Automatic Analyzer 7600‐210; Hitachi, Japan).

Fasting glucose levels were categorized as <90, 90 to 99, 100 to 109, 110 to 124, or ≥125 mg/dl. Obesity was defined as a BMI of ≥25 kg/m^2^, and abdominal obesity was defined as a waist circumference of ≥90 cm for men and ≥85 cm for women.^17^, ^18^ Uncontrolled blood pressure was defined as either SBP ≥ 140 mm Hg or DBP ≥ 90 mm Hg.^19^ Uncontrolled cholesterol levels were defined as follows: LDL‐C ≥ 130 mg/dl, TG ≥ 150 mg/dl, and HDL‐C < 40 mg/dl in men and <50 mg/dl in women.^20^

### 2.3 Statistical analysis

All analyses were performed after accounting for the complex sample design, stratification, clustering, and sample weights. We presented demographic characteristics, anthropometric variables, and laboratory variables of the study participants according to their fasting glucose levels. Mean and SE were presented for continuous variables, and unweighted numbers and weighted percentages were presented for categorical variables. To compare the prevalence over fasting glucose level groups, we calculated age‐adjusted prevalence of obesity, abdominal obesity, lifestyle factors, and metabolic parameters using direct standardization by applying standardized prevalence for the Korean population in 2005 as reference. *P* values for trends were estimated to compare the participants' characteristics according to fasting glucose levels. The relationships between fasting glucose levels and obesity, abdominal, obesity, and lifestyle factors were evaluated by multivariate logistic regression analysis. The odds ratios (ORs) and 95% confidence intervals (CIs) for obesity, abdominal obesity, and each lifestyle factor were calculated after adjusting for age and potential confounders. The associations between fasting glucose levels and the control of blood pressure and cholesterol levels were evaluated by multivariate logistic regression analysis. We presented age‐adjusted ORs with 95% CIs and multivariable adjusted ORs and 95% CIs for uncontrolled blood pressure and uncontrolled LDL‐C, TG, and HDL‐C after adjusting for age, income, education, obesity, abdominal obesity, current smoking, heavy drinking, and low physical activity. Furthermore, we plotted a restricted cubic spline of ORs for uncontrolled blood pressure and uncontrolled LDL‐C, TG, and HDL‐C using fasting glucose as continuous variables to examine the association between fasting glucose levels and poor metabolic parameters. Analyses were separately performed for men and women. Analyses were conducted with IBM SPSS Statistics for Windows version 23.0 (IBM Corp., Armonk, NY, USA), and restricted cubic splines were plotted using rms package in R software version 3.6.2 (R Foundation for Statistical Computing, Vienna, Austria) with two‐tailed *P* values <.05 considered statistically significant.

## 3 RESULTS

### 3.1 Basic characteristics of the study participants according to fasting glucose levels

Table 1 shows the demographic and metabolic characteristics according to fasting glucose levels. Age increased as fasting glucose levels increased in both men and women (*P* for trend <.001). The proportion of participants with low income and participants with ≤12 years of education increased according to fasting glucose levels in both men and women (*P* for trend <.001). As fasting glucose levels increased, height and HDL‐C decreased and weight, BMI, waist circumference, SBP, DBP, HbA1c, TC, and TG increased in both and women (*P* for trend <.05). The LDL‐C levels increased according to fasting glucose levels in women (*P* for trend <.001); however, we observed no significant association between LDL‐C and fasting glucose levels in men.

**TABLE 1:** Demographic and metabolic characteristics according to fasting glucose levels in 13 625 participants without alleged diabetes mellitus

Fasting glucose (mg/dl)	< 90	90–99	100‐109	110–124	≥ 125	*P* for trend
Men (*n* = 5912), *N*	1084	2292	1475	683	378	
Age (years)	46.6 (0.4)	48.8 (0.3)	51.7 (0.4)	53.2 (0.5)	53.3 (0.7)	<.001
Height (cm)	171.2 (0.2)	171.2 (0.2)	170.8 (0.2)	170.5 (0.3)	170.4 (0.4)	.002
Weight (kg)	68.9 (0.3)	71.5 (0.3)	73.5 (0.3)	74.7 (0.5)	76.2 (0.7)	<.001
BMI (kg/m^2^)	23.4 (0.1)	24.3 (0.1)	25.1 (0.1)	25.6 (0.1)	26.2 (0.2)	<.001
WC (cm)	83.0 (0.3)	85.7 (0.2)	87.9 (0.2)	89.8 (0.4)	91.4 (0.5)	<.001
SBP (mmHg)	115.9 (0.4)	119.0 (0.4)	123.4 (0.4)	125.8 (0.6)	127.9 (1.0)	<.001
DBP (mm Hg)	77.4 (0.3)	78.9 (0.3)	81.1 (0.3)	82.2 (0.4)	82.5 (0.7)	<.001
HbA1c (%)	5.3 (0.01)	5.5 (0.01)	5.6 (0.01)	5.9 (0.02)	7.0 (0.1)	<.001
TC (mg/dl)	193.5 (1.3)	196.1 (0.8)	198.3 (1.1)	203.7 (1.7)	204.6 (2.8)	<.001
TG (mg/dl)	156.4 (8.0)	152.0 (2.7)	182.2 (4.1)	217.4 (10.4)	248.8 (14.7)	<.001
LDL‐C (mg/dl)	113.9 (1.5)	118.3 (0.8)	115.0 (1.1)	113.4 (1.8)	110.7 (2.8)	.096
HDL‐C (mg/dl)	48.3 (0.4)	47.3 (0.3)	46.8 (0.3)	46.8 (0.5)	44.2 (0.6)	<.001
Low income	136 (9.8)	319 (9.6)	200 (10.8)	124 (14.9)	100 (21.7)	<.001
Education ≤12 years	461 (39.7)	1109 (46.1)	819 (54.1)	407 (58.6)	217 (57.7)	<.001
Women (*n* = 7713), *N*	2334	3212	1390	520	257	
Age (years)	47.7 (0.3)	51.3 (0.3)	55.5 (0.4)	60.0 (0.6)	58.1 (0.9)	<.001
Height (cm)	158.4 (0.2)	157.7 (0.1)	156.8 (0.2)	155.5 (0.3)	155.7 (0.5)	<.001
Weight (kg)	55.8 (0.2)	58.1 (0.2)	60.8 (0.3)	61.7 (0.5)	64.2 (0.9)	<.001
BMI (kg/m^2^)	22.3 (0.1)	23.3 (0.1)	24.7 (0.1)	25.5 (0.2)	26.4 (0.3)	<.001
WC (cm)	75.3 (0.2)	78.6 (0.2)	82.2 (0.3)	84.6 (0.5)	87.6 (0.7)	<.001
SBP (mm Hg)	111.0 (0.4)	116.3 (0.4)	120.5 (0.6)	125.4 (0.8)	128.1 (1.6)	<.001
DBP (mm Hg)	72.2 (0.2)	74.9 (0.2)	75.6 (0.3)	76.5 (0.5)	78.7 (0.8)	<.001
HbA1c (%)	5.3 (0.01)	5.5 (0.01)	5.7 (0.01)	6.0 (0.02)	6.9 (0.1)	<.001
TC (mg/dl)	194.3 (0.9)	197.3 (0.7)	203.2 (1.3)	205.1 (2.0)	209.4 (2.8)	<.001
TG (mg/dl)	96.6 (1.6)	113.0 (1.5)	133.0 (3.1)	146.4 (5.8)	168.7 (8.5)	<.001
LDL‐C (mg/dl)	117.9 (0.8)	120.0 (0.6)	123.8 (1.1)	124.6 (1.9)	127.2 (2.7)	<.001
HDL‐C (mg/dl)	57.1 (0.3)	54.8 (0.3)	52.8 (0.4)	51.2 (0.6)	48.6 (0.8)	<.001
Low income	336 (12.5)	598 (15.9)	330 (21.4)	161 (29.6)	79 (29.3)	<.001
Education ≤12 years	1221 (52.8)	1979 (61.7)	1015 (73.0)	401 (80.4)	200 (85.7)	<.001

*Note*: Values are presented as mean (SE) or unweighted number (weighted percentage).

Abbreviations: BMI, body mass index; DBP, diastolic blood pressure; HbA1c, glycated hemoglobin; HDL‐C, high‐density lipoprotein cholesterol; LDL‐C, low‐density lipoprotein cholesterol; SBP, systolic blood pressure; TC, total cholesterol; TG, triglycerides; WC, waist circumference.

### 3.2 Obesity, abdominal obesity, and lifestyle factors according to fasting glucose levels

Table 2 shows the relationships between fasting glucose levels and obesity, abdominal obesity, and lifestyle factors. The age‐standardized proportions of participants with obesity, abdominal obesity, and heavy drinking increased as fasting glucose levels increased in both men and women (*P* for trend <.05). We observed no significant association between smoking status and fasting glucose levels. The age‐standardized proportions of participants with low physical activity increased according to fasting glucose levels in men (*P* for trend <.001). In the multivariate model, the ORs and 95% CIs for obesity, abdominal obesity, and heavy drinking increased according to fasting glucose levels in both men and women (*P* for trend <.05). Compared to fasting glucose levels of <90 mg/dl, the odds for obesity and abdominal obesity increased from fasting glucose levels of 90 to 99 mg/dl. The odds for heavy drinking increased from fasting glucose levels of 90 to 99 mg/dl in men and 110 to 124 mg/dl in women. We observed no significant association between current smoking, low physical activity, and fasting glucose levels.

**TABLE 2:** Obesity, abdominal obesity, and lifestyle factors according to fasting glucose levels in participants without alleged diabetes mellitus

Fasting glucose (mg/dl)	< 90	90–99	100–109	110–124	≥125	*P* for trend
Men						
Obesity						
*N* (%)	288 (27.2)	838 (38.3)	713 (50.9)	379 (55.2)	206 (60.2)	<.001
Age standardized %	26.6	38.8	53.9	60.6	65.9	<.001
Crude OR (95% CI)	1.00	1.66 (1.38‐2.00)	2.79 (2.28‐3.41)	3.30 (2.61‐4.18)	4.06 (3.04‐5.43)	<.001
Age‐adjusted OR (95% CI)	1.00	1.79 (1.48‐2.17)	3.31 (2.69‐4.08)	4.12 (3.22‐5.27)	5.12 (3.81‐6.88)	<.001
aOR (95% CI)^a^	1.00	1.53 (1.22‐1.90)	2.61 (1.99‐3.43)	2.31 (1.68‐3.17)	2.24 (1.43‐3.53)	<.001
Abdominal obesity						
*N* (%)	212 (19.0)	662 (28.9)	546 (37.8)	335 (48.4)	194 (55.8)	<.001
Age standardized %	18.9	29.6	40.0	52.7	60.4	<.001
Crude OR (95% CI)	1.00	1.74 (1.41‐2.13)	2.59 (2.10‐3.19)	4.00 (3.12‐5.13)	5.40 (4.06‐7.19)	<.001
Age‐adjusted OR (95% CI)	1.00	1.78 (1.45‐2.19)	2.75 (2.22‐3.39)	4.33 (3.36‐5.57)	5.85 (4.39‐7.81)	<.001
aOR (95% CI)^b^	1.00	1.29 (1.01‐1.65)	1.44 (1.09‐1.89)	2.40 (1.75‐3.29)	3.47 (2.29‐5.24)	<.001
Current smoking						
*N* (%)	435 (41.6)	788 (37.2)	491 (37.1)	245 (38.0)	144 (40.5)	.462
Age standardized %	41.0	37.3	38.2	41.2	48.0	.108
Crude OR (95% CI)	1.00	0.83 (0.70‐0.99)	0.83 (0.69‐0.99)	0.86 (0.69‐1.08)	0.95 (0.71‐1.28)	.462
Age‐adjusted OR (95% CI)	1.00	0.88 (0.74‐1.05)	0.96 (0.79‐1.15)	1.04 (0.83‐1.32)	1.17 (0.86‐1.58)	.207
aOR (95% CI)^c^	1.00	0.83 (0.69‐1.01)	0.85 (0.69‐1.04)	0.88 (0.68‐1.15)	1.09 (0.78‐1.51)	.995
Heavy drinking						
*N* (%)	231 (20.8)	575 (25.0)	491 (33.7)	259 (38.6)	123 (33.0)	<.001
Age standardized %	21.4	25.0	34.2	38.7	32.3	.025
Crude OR (95% CI)	1.00	1.27 (1.04‐1.54)	1.93 (1.59‐2.35)	2.39 (1.89‐3.03)	1.87 (1.41‐2.49)	<.001
Age‐adjusted OR (95% CI)	1.00	1.26 (1.03‐1.53)	1.90 (1.55‐2.32)	2.34 (1.84‐2.97)	1.83 (1.37‐2.44)	<.001
aOR (95% CI)^d^	1.00	1.42 (1.15‐1.75)	2.05 (1.66‐2.54)	2.46 (1.91‐3.17)	1.96 (1.45‐2.66)	<.001
Low physical activity						
*N* (%)	611 (58.0)	1348 (60.8)	889 (62.0)	413 (61.0)	223 (63.2)	.105
Age standardized %	59.2	61.2	61.0	61.1	63.5	.001
Crude OR (95% CI)	1.00	1.12 (0.93‐1.35)	1.18 (0.98‐1.43)	1.13 (0.89‐1.43)	1.25 (0.93‐1.67)	.105
Age‐adjusted OR (95% CI)	1.00	1.08 (0.90‐1.31)	1.08 (0.89‐1.32)	1.01 (0.80‐1.28)	1.11 (0.82‐1.49)	.687
aOR (95% CI)^e^	1.00	1.09 (0.90‐1.31)	1.09 (0.90‐1.34)	0.97 (0.76‐1.25)	1.06 (0.78‐1.43)	.966
Women						
Obesity						
*N* (%)	431 (16.9)	886 (26.8)	579 (41.8)	260 (51.5)	165 (61.9)	<.001
Age standardized %	18.1	26.4	40.0	55.0	66.6	<.001
Crude OR (95% CI)	1.00	1.80 (1.55‐2.09)	3.53 (2.96‐4.22)	5.23 (4.19‐6.51)	8.00 (5.85‐10.92)	<.001
Age adjusted OR (95% CI)	1.00	1.71 (1.47‐1.99)	3.19 (2.66‐3.82)	4.46 (3.55‐5.61)	7.01 (5.09‐9.66)	<.001
aOR (95% CI)^a^	1.00	1.45 (1.19‐1.75)	2.26 (1.75‐2.91)	2.55 (1.83‐3.55)	2.44 (1.60‐3.72)	<.001
Abdominal obesity						
*N* (%)	329 (12.9)	774 (22.0)	527 (36.4)	250 (47.9)	165 (61.9)	<.001
Age standardized %	13.7	22.0	33.5	50.7	66.2	<.001
Crude OR (95% CI)	1.00	1.90 (1.62‐2.23)	3.86 (3.23‐4.61)	6.20 (4.94‐7.78)	10.95 (7.84‐15.29)	<.001
Age‐adjusted OR (95% CI)	1.00	1.73 (1.47‐2.03)	3.18 (2.65‐3.81)	4.57 (3.60‐5.80)	8.64 (6.13‐12.17)	<.001
aOR (95% CI)^b^	1.00	1.43 (1.14‐1.78)	1.87 (1.44‐2.43)	2.55 (1.82‐3.57)	4.22 (2.72‐6.56)	<.001
Current smoking						
*N* (%)	114 (5.7)	166 (5.9)	73 (6.0)	18 (3.1)	9 (3.7)	.130
Age standardized %	5.0	6.0	7.2	7.2	3.9	.870
Crude OR (95% CI)	1.00	1.05 (0.79‐1.39)	1.06 (0.75‐1.50)	0.53 (0.30‐0.92)	0.63 (0.27‐1.46)	.130
Age‐adjusted OR (95% CI)	1.00	1.61 (0.87‐1.55)	1.33 (0.93‐1.92)	0.76 (0.43‐1.36)	0.87 (0.37‐2.03)	.770
aOR (95% CI)^c^	1.00	1.09 (0.80‐1.49)	1.23 (0.82‐1.84)	0.61 (0.33‐1.13)	0.46 (0.15‐1.41)	.338
Heavy drinking						
*N* (%)	219 (11.0)	332 (11.2)	150 (11.2)	59 (11.9)	24 (11.4)	.699
Age standardized %	9.7	12.3	13.7	16.1	14.0	.013
Crude OR (95% CI)	1.00	1.01 (0.82‐1.25)	1.02 (0.79‐1.31)	1.09 (0.77‐1.54)	1.04 (0.63‐1.71)	.699
Age adjusted OR (95% CI)	1.00	1.15 (0.93‐1.43)	1.36 (1.05‐1.77)	1.74 (1.21‐2.51)	1.55 (0.93‐2.59)	.001
aOR (95% CI)^d^	1.00	1.10 (0.89‐1.37)	1.25 (0.94‐1.66)	1.65 (1.13‐2.42)	1.40 (0.81‐2.41)	.011
Low physical activity						
*N* (%)	1434 (63.2)	2005 (63.6)	887 (65.3)	354 (72.7)	183 (74.6)	<.001
Age standardized %	63.7	64.3	61.9	63.4	71.6	.170
Crude OR (95% CI)	1.00	1.02 (0.89‐1.16)	1.09 (0.93‐1.29)	1.55 (1.23‐1.95)	1.71 (1.20‐2.44)	<.001
Age‐adjusted OR (95% CI)	1.00	0.96 (0.84‐1.09)	0.95 (0.81‐1.13)	1.26 (1.00‐1.60)	1.44 (1.01‐2.06)	.086
aOR (95% CI)^e^	1.00	0.95 (0.83‐1.08)	0.93 (0.78‐1.10)	1.19 (0.93‐1.51)	1.35 (0.94‐1.94)	.281

Abbreviations: aOR, adjusted odds ratio; CI, confidence interval; OR, odds ratio.

^a^ aORs (95% CI) were calculated after adjusting for age, income, education, abdominal obesity, current smoking, heavy drinking, and low physical activity.

^b^ aORs (95% CI) were calculated after adjusting for age, income, education, obesity, current smoking, heavy drinking, and low physical activity.

^c^ aORs (95% CI) were calculated after adjusting for age, income, education, obesity, abdominal obesity, heavy drinking, and low physical activity.

^d^ aORs (95% CI) were calculated after adjusting for age, income, education, obesity, abdominal obesity, current smoking, and low physical activity.

^e^ aORs (95% CI) were calculated after adjusting for age, income, education, obesity, abdominal obesity, current smoking, and heavy drinking.

### 3.3 Control of blood pressure and cholesterol according to fasting glucose levels

Table 3 shows the associations between fasting glucose levels and the control of blood pressure and cholesterol levels. The age‐standardized proportions of participants with BP ≥140/90 mm Hg increased as fasting glucose levels increased in both men and women (*P* for trend <.001). As for the control of cholesterol levels, the age‐standardized proportions of participants with TG ≥ 150 mg/dl and HDL‐C < 40 mg/dl increased according to the fasting glucose levels in men, and the age‐standardized proportions of participants with LDL‐C ≥ 130 mg/dl, TG ≥ 150 mg/dl, and HDL‐C <50 mg/dl increased according to the fasting glucose levels in women (*P* for trend <.001). In the multivariate model, the ORs and 95% CIs for BP ≥140/90 mm Hg increased as fasting glucose levels increased in both men and women (*P* for trend <.001). Compared to fasting glucose levels of <90 mg/dl, the increase was significant from fasting glucose levels of 90 to 99 mg/dl. Furthermore, as for control of cholesterol levels among men, the ORs and 95% CIs for TG ≥150 mg/dl and HDL < 40 mg/dl increased according to fasting glucose levels (*P* for trend <.05). Compared to fasting glucose levels of <90 mg/dl, the odds for TG ≥ 150 mg/dl increased from fasting glucose levels of 100 to 109 mg/dl whereas that for HDL < 40 mg/dl increased only at fasting glucose levels of ≥125 mg/dl. In women, the ORs and 95% CIs for LDL‐C ≥ 130 mg/dl, TG ≥150 mg/dl and HDL‐C < 50 mg/dl all increased according to fasting glucose levels (*P* for trend <.05). Compared to fasting glucose levels of <90 mg/dl, the odds for LDL‐C ≥ 130 mg/dl increased from fasting glucose levels of 100 to 109 mg/dl whereas those for TG ≥ 150 mg/dl and HDL‐C < 50 mg/dl increased from fasting glucose levels of 90 to 99 mg/dl.

**TABLE 3:** Control of blood pressure and cholesterol according to fasting glucose levels in participants without alleged diabetes mellitus

Fasting glucose (mg/dl)	<90	90–99	100–109	110–124	≥125	*P* for trend
Men						
BP ≥ 140/90 mm Hg						
*N* (%)	136 (12.1)	379 (16.6)	358 (23.8)	199 (29.9)	119 (33.8)	<.001
Age standardized %	12.4	16.2	24.0	29.7	32.5	<.001
Crude OR (95% CI)	1.00	1.45 (1.12‐1.87)	2.27 (1.78‐2.90)	3.11 (2.35‐4.11)	3.71 (2.71‐5.09)	<.001
Age‐adjusted OR (95% CI)	1.00	1.43 (1.11‐1.85)	2.20 (1.72‐2.82)	2.99 (2.25‐3.98)	3.57 (2.58‐4.93)	<.001
aOR (95% CI)^a^	1.00	1.34 (1.03‐1.76)	1.82 (1.41‐2.35)	2.32 (1.70‐3.16)	2.80 (1.97‐4.00)	<.001
LDL‐C ≥ 130 mg/dl						
*N* (%)	351 (33.4)	806 (36.7)	485 (33.1)	241 (25.0)	127 (34.9)	.881
Age standardized %	33.0	36.4	34.3	36.9	36.5	.092
Crude OR (95% CI)	1.00	1.15 (0.95‐1.40)	0.99 (0.80‐1.21)	1.08 (0.85‐1.37)	1.07 (0.79‐1.45)	.881
Age‐adjusted OR (95% CI)	1.00	1.18 (0.97‐1.43)	1.03 (0.84‐1.26)	1.14 (0.90‐1.45)	1.13 (0.84‐1.53)	.646
aOR (95% CI)^a^	1.00	1.22 (1.00‐1.49)	1.13 (0.91‐1.40)	1.22 (0.94‐1.57)	1.33 (0.96‐1.83)	.144
TG ≥ 150 mg/dl						
*N* (%)	348 (32.3)	815 (36.5)	666 (48.4)	358 (54.9)	221 (62.0)	<.001
Age standardized %	32.9	37.0	49.2	56.3	64.1	<.001
Crude OR (95% CI)	1.00	1.20 (1.01‐1.43)	1.97 (1.64‐2.36)	2.55 (2.02‐3.22)	3.43 (2.59‐4.53)	<.001
Age‐adjusted OR (95% CI)	1.00	1.26 (1.05‐1.50)	2.19 (1.81‐2.65)	2.92 (2.30‐3.71)	3.96 (2.97‐5.27)	<.001
aOR (95% CI)^a^	1.00	1.14 (0.95‐1.37)	1.85 (1.51‐2.28)	2.21 (1.71‐2.85)	2.99 (2.20‐4.06)	<.001
HDL‐C < 40 mg/dl						
*N* (%)	244 (22.2)	558 (23.9)	428 (27.8)	203 (28.6)	133 (37.8)	<.001
Age standardized %	22.5	24.0	29.1	30.0	39.6	<.001
Crude OR (95% CI)	1.00	1.10 (0.90‐1.35)	1.35 (1.10‐1.66)	1.41 (1.09‐1.83)	2.13 (1.56‐2.91)	<.001
Age‐adjusted OR (95% CI)	1.00	1.10 (0.90‐1.34)	1.33 (1.08‐1.64)	1.39 (1.07‐1.80)	2.09 (1.53‐2.86)	<.001
aOR (95% CI)^a^	1.00	0.98 (0.79‐1.21)	1.16 (0.93‐1.47)	1.13 (0.84‐1.52)	1.63 (1.16‐2.29)	.006
Women						
BP ≥ 140/90 mm Hg						
*N* (%)	201 (7.3)	479 (13.4)	287 (17.8)	127 (22.5)	67 (28.2)	<.001
Age standardized %	8.1	12.0	14.1	19.9	25.7	<.001
Crude OR (95% CI)	1.00	1.97 (1.59‐2.44)	2.75 (2.18‐3.47)	3.68 (2.77‐4.90)	4.99 (3.47‐7.17)	<.001
Age‐adjusted OR (95% CI)	1.00	1.68 (1.36‐2.08)	1.93 (1.52‐2.45)	2.13 (1.57‐2.90)	3.24 (2.18‐4.80)	<.001
aOR (95% CI)^a^	1.00	1.57 (1.26‐1.97)	1.73 (1.35‐2.23)	1.81 (1.30‐2.53)	2.49 (1.63‐3.79)	<.001
LDL‐C ≥ 130 mg/dl						
*N* (%)	762 (32.3)	1151 (35.1)	552 (40.9)	217 (43.0)	110 (47.1)	<.001
Age standardized %	31.9	33.0	37.2	40.8	46.8	<.001
Crude OR (95% CI)	1.00	1.13 (0.98‐1.31)	1.45 (1.21‐1.73)	1.58 (1.25‐1.99)	1.86 (1.40‐2.49)	<.001
Age‐adjusted OR (95% CI)	1.00	1.06 (0.92‐1.23)	1.26 (1.06‐1.52)	1.27 (1.00‐1.62)	1.56 (1.15‐2.10)	<.001
aOR (95% CI)^a^	1.00	1.04 (0.89‐1.20)	1.20 (1.00‐1.45)	1.29 (1.01‐1.65)	1.42 (1.02‐1.97)	.004
TG ≥ 150 mg/dl						
*N* (%)	305 (11.7)	649 (19.1)	400 (28.2)	168 (32.2)	119 (46.5)	<.001
Age standardized %	12.8	18.4	27.3	38.9	48.9	<.001
Crude OR (95% CI)	1.00	1.78 (1.49‐2.13)	2.97 (2.44‐3.62)	3.59 (2.78‐4.64)	6.59 (4.76‐9.13)	<.001
Age‐adjusted OR (95% CI)	1.00	1.67 (1.40‐2.00)	2.59 (2.11‐3.18)	2.90 (2.21‐3.80)	5.55 (3.97‐7.74)	<.001
aOR (95% CI)^a^	1.00	1.49 (1.24‐1.79)	1.99 (1.61‐2.46)	2.06 (1.54‐2.76)	3.66 (2.53‐5.29)	<.001
HDL‐C < 50 mg/dl						
*N* (%)	684 (29.1)	1243 (36.9)	621 (42.8)	268 (49.6)	148 (59.2)	<.001
Age standardized %	28.9	36.8	41.8	58.8	59.8	<.001
Crude OR (95% CI)	1.00	1.43 (1.25‐1.63)	1.83 (1.55‐2.15)	2.40 (1.93‐2.97)	3.53 (2.57‐4.86)	<.001
Age‐adjusted OR (95% CI)	1.00	1.33 (1.17‐1.52)	1.58 (1.33‐1.87)	1.91 (1.53‐2.37)	2.93 (2.12‐4.06)	<.001
aOR (95% CI)^a^	1.00	1.29 (1.12‐1.48)	1.33 (1.11‐1.59)	1.65 (1.30‐2.09)	2.22 (1.55‐3.20)	<.001

Abbreviations: aOR, adjusted odds ratio; BP, blood pressure; CI, confidence interval; HDL‐C, high‐density lipoprotein cholesterol; LDL‐C, low‐density lipoprotein cholesterol; OR, odds ratio; TG, triglycerides.

^a^ aORs (95% CI) were calculated after adjusting for age, income, education, obesity, abdominal obesity, current smoking, heavy drinking, and low physical activity.

**FIGURE 1:**
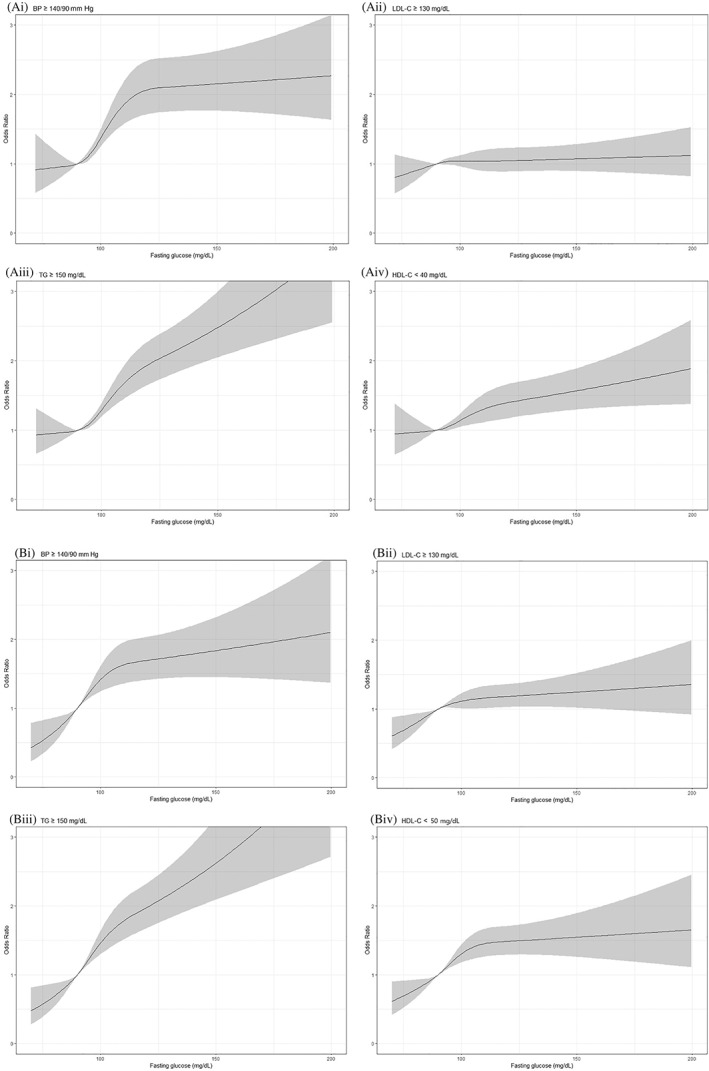
Fasting glucose and risk of poor metabolic parameters. (A) Men. (i) BP ≥140/90 mm Hg. (ii) LDL‐C ≥ 130 mg/dl. (iii) TG ≥150 mg/dl. (iv) HDL‐C < 40 mg/dl. (B) Women. (i) BP ≥140/90 mm Hg. (ii) LDL‐C ≥ 130 mg/dl. (iii) TG ≥150 mg/dl. (iv) HDL‐C < 50 mg/dl. BP, blood pressure; HDL‐C, high‐density lipoprotein cholesterol; LDL‐C, low‐density lipoprotein cholesterol; TG, triglycerides

Figure 1 shows the spline representation for the association between fasting glucose levels and poor metabolic parameters. After using fasting glucose as a continuous variable, compared to fasting glucose 90 mg/dl, the odds for BP ≥ 140/90 mm Hg, TG ≥ 150 mg/dl, HDL‐C < 40 mg/dl in men, and HDL‐C < 50 mg/dl in women increased as fasting glucose increased. The increases in odds for poor metabolic parameters were observed even in normal fasting glucose range.

## 4 DISCUSSION

In this nationwide cross‐sectional study, obesity, abdominal obesity, and heavy drinking were modifiable lifestyle risk factors that were associated with higher fasting glucose levels in individuals without previous diagnosis of diabetes. Higher fasting glucose levels were associated with poor control of blood pressure, TGs, and HDL‐C in both men and women. Furthermore, higher fasting glucose levels were associated with poor LDL‐C in women. Notably, the odds for poor control of these metabolic profiles mostly increased from the fasting glucose ranges of 90 to 99 mg/dl and above.

Higher fasting glucose levels in individuals without previous diagnosis of diabetes were associated with poor blood pressure control in our study. The association between diabetes or IFG and hypertension has been well established; however, to our knowledge, only two studies have shown a linear association between different ranges of fasting glucose and blood pressure.^21^, ^22^ A prospective cohort study of healthy middle‐aged men in Norway reported a linear association between fasting glucose level and resting and exercise blood pressures and the development of hypertension.^21^ Furthermore, a Japanese study observed that the cumulative incidence of hypertension was positively correlated with increased baseline fasting glucose levels.^22^ Although most other studies investigated the association between IFG or diabetes and hypertension, these studies considered the pre‐IFG range of fasting glucose level as a baseline and showed increased risks of high blood pressure for higher fasting glucose levels. Similarly, in our study, we observed that the odds of uncontrolled blood pressure increased for fasting glucose levels of 90 to 99 mg/dl compared to <90 mg/dl, suggesting an elevated risk for high blood pressure within the normal fasting glucose level range. High blood glucose levels reflect hyperinsulinemia and insulin resistance. Increased renal sodium retention and enhanced sympathetic nervous system activity caused by hyperinsulinemia may contribute to increased blood pressure.^23^, ^24^ Furthermore, chronic activation of the sympathetic nervous system may lead to a further increase of insulin resistance, which also contributes to the development of diabetes and hypertension.^23^, ^24^

Higher fasting glucose levels were associated with uncontrolled lipid profiles in our analysis; specifically, uncontrolled TG and HDL‐C in men and uncontrolled LDL‐C, TG, and HDL‐C in women. Previous studies also demonstrated the associations between IFG and poor lipid profiles.^11^, ^25^ Our results additionally showed that higher fasting glucose levels within the pre‐IFG range were also associated with uncontrolled TG and HDL‐C levels and that the risk for poor control of lipids linearly increased with increasing fasting glucose levels. A study in Taiwan showed an association between elevated fasting glucose levels within the pre‐IFG range and an increased risk for metabolic syndrome in older women.^26^ In that study, women with fasting glucose levels of 95 to 99 mg/dl had higher odds for hypertriglyceridemia and low HDL‐C compared to women with fasting glucose levels of <90 mg/dl; moreover, TC, TG, and HDL‐C levels were significantly correlated with fasting glucose levels.^26^ The main cause of diabetic dyslipidemia is insulin resistance.^27^ Poor lipid profiles among individuals with higher fasting glucose levels in our study may reflect the development of insulin resistance. Insulin resistance reduces the suppression of lipolysis in adipose tissue, increases very low‐density lipoprotein secretion, and contributes to low HDL‐C and hypertriglyceridemia.^28^ Furthermore, the common mediators of dyslipidemia and dysglycemia, such as tumor necrosis factor, may alter lipid profiles.^29^ The observed increasing trends of poor lipid profiles according to fasting glucose levels were prominent in women in our study. Similarly, a Chinese study observed associations between IFG and plasma lipids concentration only in women.^25^ More attention should be given to women with higher fasting glucose levels as they may present with accompanying dyslipidemia.

Among modifiable risk factors for diabetes, obesity, abdominal obesity, and heavy drinking were positively correlated with fasting glucose levels. Obesity or abdominal obesity often coexists with diabetes as they are linked with defective insulin secretion and insulin resistance.^30^ An increase in overall fatness, especially visceral fat, contributes to insulin resistance.^30^ Despite the well‐established relationship between obesity and diabetes, the prevalence of obesity and abdominal obesity among Korean diabetes patients exceeded 50%,^2^ indicating that more than half of the diabetes patients still live with modifiable risk factors for diabetes. Efforts should be undertaken to prevent obesity, and individuals with obesity or abdominal obesity should be screened for high blood glucose levels.

The relationship between alcohol consumption and type 2 diabetes can be illustrated by a J‐shaped curve. A recent meta‐analysis reported that, compared to abstainers, the risk for type 2 diabetes decreased for <63 g/day and increased above this threshold.^9^ Excessive alcohol consumption can disturb glucose hemostasis by inhibiting insulin secretion and causing whole‐body insulin resistance.^31^ In our study, heavy drinking was associated with increased fasting glucose levels in both men and women. Individuals who consume excessive alcohol should reduce their alcohol intake to improve their blood glucose control.^32^

This study has several limitations. The major limitation was its cross‐sectional design. Although our results demonstrated the positive associations between fasting glucose level and several lifestyle factors and metabolic parameters among adults without diagnosis of diabetes, causal interpretation of these findings is difficult. Furthermore, recall bias may have influenced the classifications of lifestyle factors in this study. Despite these limitations, our study included a large number and nationally representative sample of Korean adults, which enabled us to convey the public health implications regarding the relationship between fasting glucose levels with multiple lifestyle factors and metabolic parameters among individuals who are not treated for diabetes.

In conclusion, higher fasting glucose levels in individuals without diagnosis of diabetes were associated with poor blood pressure control and lipid profiles even in the pre‐IFG range of fasting glucose levels. Obesity, abdominal obesity, and heavy drinking were associated with higher fasting glucose levels in individuals without diagnosis of diabetes. Efforts should be undertaken to prevent increased fasting glucose levels, as higher levels, even within the normal range, were associated with poor metabolic profiles. Furthermore, individuals with fasting glucose levels of 90 to 99 mg/dl should also be screened for hypertension and dyslipidemia as they are more likely to have worse metabolic profiles than those with levels of <90 mg/dl. Lifestyle interventions, especially the prevention of obesity, abdominal obesity, and heaving drinking, should be emphasized to prevent increased fasting glucose levels.

## DISCLOSURE

The authors declare that there is no competing interest.
